# Municipal Sewage Sludge as a Source for Obtaining Efficient Biosorbents: Analysis of Pyrolysis Products and Adsorption Tests

**DOI:** 10.3390/ma16072648

**Published:** 2023-03-27

**Authors:** Krzysztof Mazurek, Sebastian Drużyński, Urszula Kiełkowska, Adam Węgrzynowicz, Anna K. Nowak, Zbigniew Wzorek, Adriana Wróbel-Kaszanek

**Affiliations:** 1 Faculty of Chemistry, Nicolaus Copernicus University in Toruń, 7 Gagarin Street, 87-100 Toruń, Poland; 2 Faculty of Chemical Engineering and Technology, Cracow University of Technology, 24 Warszawska Street, 31-155 Kraków, Poland

**Keywords:** sewage sludge, biochar, gas, tar, wastewater, adsorption, removal, Cu^2+^, Zn^2+^

## Abstract

In the 21st century, the development of industry and population growth have significantly increased the amount of sewage sludge produced. It is a by-product of wastewater treatment, which requires appropriate management due to biological and chemical hazards, as well as several legal regulations. The pyrolysis of sewage sludge to biochar can become an effective way to neutralise and use waste. Tests were carried out to determine the effect of pyrolysis conditions, such as time and temperature, on the properties and composition of the products obtained and the sorption capacity of the generated biochar. Fourier transform infrared analysis (FTIR) showed that the main components of the produced gas phase were CO_2_, CO, CH_4_ and to a lesser extent volatile organic compounds. In tar, compounds of mainly anthropogenic origin were identified using gas chromatography mass spectrometry (GC-MS). The efficiency of obtaining biochars ranged from 44% to 50%. An increase in the pyrolysis temperature resulted in a decreased amount of biochar produced while improving its physicochemical properties. The biochar obtained at high temperatures showed the good adsorption capacity of Cu^2+^ (26 mg·g^−1^) and Zn^2+^ (21 mg·g^−1^) cations, which indicates that it can compete with similar sorbents. Adsorption of Cu^2+^ and Zn^2+^ proceeded according to the pseudo-second-order kinetic model and the Langmuir isotherm model. The biosorbent obtained from sewage sludge can be successfully used for the separation of metal cations from water and technological wastewater or be the basis for producing modified and mixed carbon sorbents.

## 1. Introduction

The 21st century has brought many challenges to humanity, and climate change seems to be among the most important ones. To a large extent, climate change is anthropogenic in nature and is a consequence of the development of industry, technology and science, as well as the growth of the world’s human population. The increase in production and population is inextricably linked to the emission and production of larger amounts of solid, liquid and gaseous waste. This is particularly evident in urban agglomerations, where population growth is accompanied by an increased amount of municipal and sewage waste [1,2,3].

Sewage sludge is a typical waste product generated in areas inhabited by people. It is the product of the municipal wastewater treatment process, which contains valuable organic matter and many pollutants that are dangerous to the environment and people. These include pathogens, heavy metals, polycyclic aromatic hydrocarbons, dioxins and pharmaceuticals. Some of the compounds contained in sewage sludge have carcinogenic, teratogenic and genotoxic properties. These pollutants can get into soils and groundwater along with precipitation [4,5,6,7,8,9,10,11].

**Figure 1:**
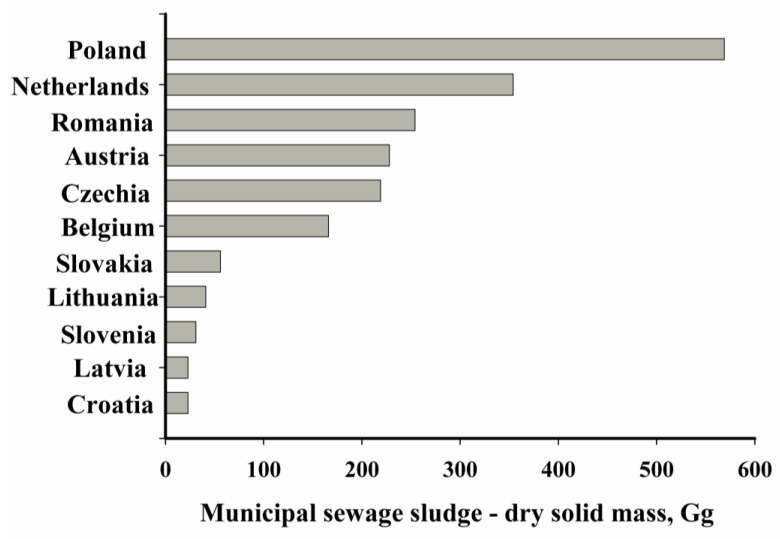
Sewage sludge production volume from municipal wastewater in dry substances in selected EU countries in 2020 [12].

The amount of sewage sludge produced is increasing year by year. This is caused by the growing human population, and also the modernisation of treatment plants to adapt them to high-efficiency technologies aimed at removing biogenic compounds, the expansion of the sewage system, the increasing percentage of urban and rural populations serviced by treatment plants and the construction of new sewage treatment plants. In China, the amount of generated waste of this type increased from 5 million Mg in 2004 to approximately 80 million Mg in 2020. The situation on the European continent is similar. In 2010, approximately 11 million Mg of dry sewage sludge matter was produced in European Union (EU) countries; by 2020 this value had increased to 13 million Mg. In Poland, the production of sewage sludge has been at a constant level for years, oscillating between 550,000 and 600,000 Mg of dry matter per year (Figure 1) [4,5,6,7,8,9,10,11].

In connection with this, new and comprehensive methods of conversion of this type of waste are intensively sought. One of the key problems is the significant differences in the properties and composition of sewage sludge. For example, this product in China contains 30% to 50% *w*/*w* organic matter, reaching 60% to 70% *w*/*w* in EU27 [4,13,14]. Sewage sludge is classified as waste, which, under Directive 2018/851/EC, should be handled in line with the established hierarchy. Sewage sludge is usually discussed in the context of its management so that energy and various substances or materials can be obtained from them to be used for other purposes [15,16].

Among the sewage sludge management methods under consideration is the pyrolysis process. During thermal decomposition in a temperature range of 300–800 °C in anaerobic conditions, organic matter decomposes into three types of products: pyrolysis oil, gas and carbon residue, that is, biochar. The main factors affecting the decomposition and properties of the obtained products are the final temperature of the process, the heating rate of the raw material and the composition of sewage sludge. Accurate control of these parameters makes it possible to obtain the desired products of appropriate quality [17,18,19,20]. Most of the work published so far has focused on obtaining pyrolysis oil with a higher calorific value. However, this process requires additional activities and modifications owing to the high content of heteroatoms and high viscosity. To a lesser extent, the research focused on the obtained biochar, which could become a material used in gas and liquid purification processes or be the basis for obtaining modified carbon materials [21,22,23,24].

The development of a cheap and effective adsorbent for the adsorption of pollutants from gases or water solutions is currently a major challenge for materials engineering. There are high hopes for biochar in this regard. This material is characterised by high porosity and many functional groups present on its surface. It can be made from organic waste materials, which makes it a cheaper sorbent than activated carbons or oxide sorbents [25,26,27,28,29,30,31].

This article describes a one-stage method of pyrolysis of sewage sludge from a medium-sized municipal sewage treatment plant. The effect of temperature and time on the structure, the surface morphology, the elemental composition of the obtained carbon material and the composition of other products was determined. In addition, the sorption capacity of the obtained biochar was tested in relation to Cu^2+^ and Zn^2+^ ions. The study described in the article is interesting and important for developing a new, easily available and cheap sorption material. Moreover, the results presented in the paper will allow us to determine the possibilities and plan the method of modifying the surface of the obtained biochars in order to improve their sorption properties. Undertaking this study is additionally justified by the use for this purpose of sewage sludge, a waste that requires new and comprehensive ways of management.

## 2. Materials and Methods

### 2.1. Reagents and Apparatus

The dried sewage sludge used in this study was collected from TW Tarnów, Poland. Reagents used in the tests (CuSO_4_∙5H_2_O, ≥99.0 wt%, and ZnSO_4_∙7H_2_O, ≥99.0 wt%) were purchased from Chempur, Poland.

The pyrolysis products identification and characterisation were carried out by:

- Quanta 3D FEG dual beam scanning electron microscope produced by FEI Company (Hillsboro, OR, USA) (HR–SEM);
- Scanning electron microscope model 1430 VP produced by LEO Electron Microscopy Ltd. (Cambridge, UK) with energy dispersive X-ray spectrometer Quantax 200 produced by Bruker AXS (Berlin, Germany) (SEM–EDX);
- Netzsch Thermoanalyzer Jupiter STA 449 F5 (Selb, Germany) with autosampler combined with FTIR spectrometer Vertex 70V by Bruker Optik (Ettlingen, Germany) (TGA-DTA with FTIR);
- Micromeritics ASAP 2020 (Norcross, GA, USA) adsorption analyser (A_BET_);
- Malvern Panalytical Zetasizer Nano-ZS ZEN3600 (Malvern, UK) (electrophoretic mobility).

The adsorption experiments were carried out in the thermostated bath for an appropriate period with continuous magnetic stirring. A constant temperature was maintained by applying the Polystat CC1 thermorelay with an accuracy of ±0.1 °C. 

The concentration of Cu^2+^ and Zn^2+^ ions in the solutions and the qualitative analysis of the liquid and solid phase was determined using the EDXRF method. The MiniPal 4 PANalytical (Almelo, The Netherlands) compact energy dispersive X-ray spectrometer was employed in the investigations. The quantitative analysis of the mineralised sewage sludge sample was conducted using AAnalyst 300 atomic absorption spectrometer produced by Perkin Elmer (Waltham, MA, USA). 

### 2.2. Experimental Pyrolysis Procedures

**Figure 2:**
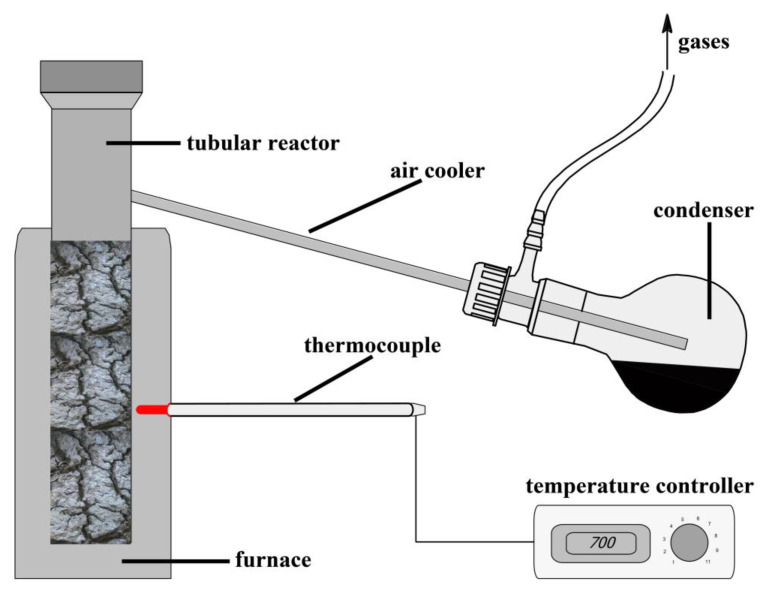
Diagram of pyrolysis reactor set.

The pyrolysis experiments were obtained without air access at a temperature range of 400–700 °C in the reactor set presented in Figure 2. The reactor was heated to a set temperature at a 10 °C·min^−1^ heating rate. After it reached the set temperature, the process was continued for 1 to 5 h. After this time, the heating was turned off, and the entire reactor content was allowed to cool down. A round-bottomed flask collected the pyrolytic tar at the tail of the cooling unit. Pyrolysis gases were collected in the calibrated glass bottle. The biochar was weighed after the complete cooling of the reactor. The obtained solid products were ground in a planetary ball mill. They were then characterised according to their physicochemical and adsorption properties. As a result of the pyrolysis processes, eight biochars were obtained under pyrolysis conditions, which are presented in Table 1.

### 2.3. The Adsorption Procedure

The adsorption study was conducted with the use of standard solutions of Cu^2+^ and Zn^2+^ with concentrations from 100 to 400 mg·dm^−3^. Sorption conditions were changed depending on the studied parameters: pH range of 1–5, adsorption temperature 20–50 °C, and contact time of 15–360 min. The detailed description of the adsorption procedure was published elsewhere [28,29]. 

The adsorption efficiency of the studied ions was determined using the equation:

(1) $$A\;[\%]=\frac{c_{0}-c_{e}}{c_{0}}\cdot 100\%$$

where *c*_0_, *c_e_* are the concentrations of Cu^2+^ or Zn^2+^ ions (mg·dm^−3^) in aqueous phase before and after adsorption process, respectively.

Moreover, the kinetics of the adsorption process was studied. For this purpose, adsorption was conducted with the use of the appropriate amount of adsorbent and Cu^2+^ or Zn^2+^ solutions at 300 mg∙dm^−3^, in 15–360-min intervals, at a temperature of 40 °C. The equilibrium capacity of the adsorbent *q_e_* (mg∙g^−1^) was calculated according to Equation (2):

(2) $$q_{e}=\frac{\left(c_{0}-c_{e}\right)\cdot V}{m}$$

where *V*—volume of solution, *m*—mass of adsorbent.

Among the different models that can be employed to describe the kinetics of ion adsorption, the pseudo-first order (PFO—Equation (3)), the pseudo-second order (PSO—Equation (4)), and the intraparticle diffusion model (IPD—Equation (5)) were selected. These equations are the most frequently used theoretical models [32,33].

(3) $$\log\left(q_{e}-q_{t}\right)=\log q_{e}-k_{1}\cdot t$$

(4) $$\frac{t}{q_{t}}=\frac{1}{k_{2}\cdot q_{e}^{2}}+\frac{1}{q_{e}}\cdot t$$

(5) $$q_{t}=k_{i}\cdot t^{1/2}$$

where *q_e_* and *q_t_* define the amounts of metal ions adsorbed at the equilibrium point and after time *t* (min), respectively, and *k*_1_ (min^−1^) is the rate constant for the pseudo-first-order model; *k*_2_ (g·mg^−1^·min^−1^) is the rate constant for the pseudo-second-order model; *k_i_* denotes the intraparticle diffusion rate constant (mg·g^−1^·min^−1/2^).

To determine the relationship between the concentrations of absorbed Cu^2+^ and Zn^2+^ ions and those concentrated in the liquid phase at equilibrium, isotherm studies were carried out. To estimate the adsorption isotherms of Cu^2+^ and Zn^2+^ ions onto the studied biochar, Freundlich (Equation (6)), Langmuir (Equation (7)), and Temkin (Equation (8)) models were used to fit the experimental data [34,35].

(6) $$q_{e}=K_{F}\cdot c_{e}^{1/n}$$

(7) $$\frac{c_{e}}{q_{e}}=\frac{1}{K\cdot q_{m}}+\frac{c_{e}}{q_{m}}$$

(8) $$q=B\cdot lnA_{T}+B\cdot lnc_{e}$$

where *q_e_* (mg·g^−1^)—real adsorption at sorption equilibrium, *c_e_* (mg·dm^−3^)—equilibrium concentration of cations, *K_F_* (mg·g^−1^)—the Freundlich constant, 1/*n*—characteristic constant related to the energy heterogeneity of the adsorbent surface, *q_m_* (mg·g^−1^)—the maximum monolayer capacity, *K* (dm^3^·mg^−1^)—the Langmuir constant, *A_T_* (dm^3^·g^−1^)—the equilibrium binding constant, and *B* = $\frac{RT}{b_{T}}$ (J·mol^−1^)—the constant related to the heat of sorption, *b_T_*—the adsorption constant.

The error functions were employed to evaluate the fit of the isotherm to the experimental equilibrium data. In this study, the residual root mean square error (*RMSE*—Equation (9)), the chi-square test (Equation (10)) and the linear regression were used [36,37]:

(9) $$RMSE=\sqrt{\frac{1}{n-2}\sum_{i-1}^{n}{\left(q_{e,exp}-q_{e,cal}\right)}^{2}}$$

(10) $$\chi^{2}=\sum_{i=1}^{n}\frac{{\left(q_{e,exp}-q_{e,cal}\right)}^{2}}{q_{e,exp}}$$

where *n* is the number of observations in the experimental isotherm, and *q_e,exp_* and *q_e,cal_* stand for experimental and calculated values, respectively. The better the curve fitting, the smaller the *RMSE* and *χ*^2^ values.

### 2.4. Analytical Methods

The EDXRF method was used to determine the concentrations of Cu^2+^ and Zn^2+^ ions in the solutions [38]. Each measurement was performed three times and then averaged. 

## 3. Results

### 3.1. Sewage Sludge Characterisation

Municipal sewage sludge, which is a waste product of chemical and biological wastewater treatment, is a complex material consisting of water, organic matter, inorganic substances, pathogenic microorganisms and mechanical impurities. Organic matter, composed mainly of particles of undigested substances, is a mixture consisting primarily of proteins, lipids and polysaccharides, which make up about 80% of the total organic carbon content. The rest are humic substances, deoxyribonucleic acids and uric acids. Inorganic components are mainly anthropogenic in nature, but can also come from soil or sewage systems. On the other hand, water, which is the main quantitative component of sewage sludge, can occur in a free and colloidally, biologically and capillary bound form. Free water, also called gravitational water, which makes up more than 60% of the total amount of water, can be easily removed by a simple mechanical dewatering method in gravity or flotation thickeners. Water bound colloidally and by the forces of adhesion and cohesion, constituting between 10% and 25%, can be partially removed with mechanical dewatering devices. Biologically bound water, whose quantitative share is the smallest, can be removed by destroying the biological membrane in the sludge disintegration processes using thermal, mechanical, chemical and other methods [39,40]. 

Depending on the processes and operations used, sewage sludge may contain on average 50% *w*/*w* to 70% *w*/*w* organic matter in dry matter. The dried sewage sludge also contains significant amounts of minerals, the content of which may vary from 30% to up to 50% *w*/*w* [41,42]. Table 2 presents the results of the quantitative analysis of mineral components of the tested sewage sludge. For this purpose, the sewage sludge sample was ground and mineralised in an open system in sulfuric acid (1:4) in Kjeldahl flasks and aqua regia, then subjected to quantitative analysis using atomic absorption spectrometry. In Table 2, the obtained results are compared with those published by Gao et al. [43]. The main mineral components of the examined sludge are P, K, Ca, Si, Mg, Na, Fe, Al and Zn, and trace amounts of Cu, Ti, Cr, Ni, Cd and Pb. The tested sewage sludge was characterised by a pH between 7.0 and 7.5, that is, it had a neutral to slightly alkaline reaction.

### 3.2. The Effects of the Pyrolysis Process Conditions on the Number of Products Obtained

Table 3 shows the results of tests performed to determine the effect of temperature on the number of pyrolysis products obtained. These data clearly indicate that a rise in temperature increases the efficiency of obtaining liquid and gaseous products. The yield of biochar decreased from 54.3% at 400 °C to 44.3% at 700 °C. The smallest effect of temperature is observed in the case of released gases. At 400 °C, the share of gaseous products was just over 13%. However, at the highest temperature tested, it increased to nearly 17%. The efficiency of tar production in the case of the tested process ranged between 33% and 39%.

### 3.3. Physical and Chemical Properties of Pyrolysis Products

**Figure 3:**
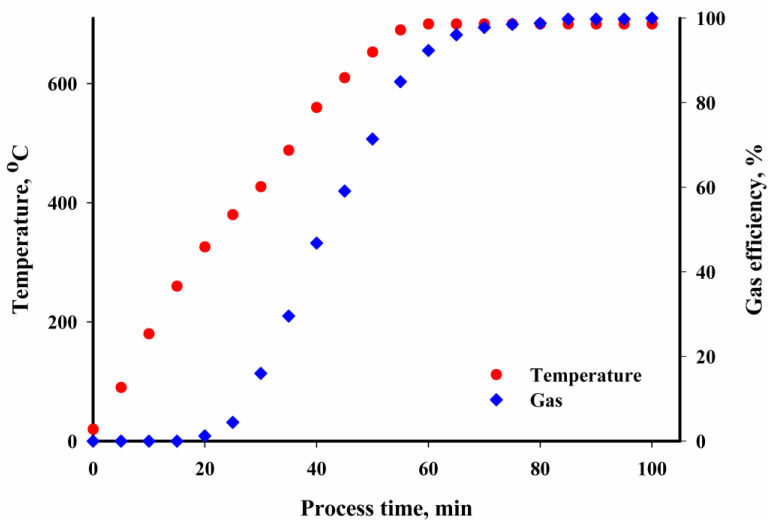
Dynamics of the gas evolution process.

The pyrolysis gas evolved at temperatures ranging from 300 to 700 °C, confirmed by Figure 3 which shows an example of the dynamics of the gas evolved depending on the process temperature. The largest increase in gas volume was observed when the temperature increased above 400 °C. Below this temperature, the dynamics of gas evolution were very slow. Analysis of the gas composition in the Orsat gas analyser showed that the increased process temperature decreased the amount of carbon oxides evolved (Table 4). The amount of energy-valuable methane increased. The remaining volume consisted of hydrocarbons with carbon atoms C_2_ and C_3_, the amount of which also rose with the increased process temperature, and NH_3_, HCN, H_2_S and CH_3_S contaminating the gas, as well as water vapour and nitrogen. Similar results were obtained by Gao et al. [43] and Djandja et al. [44], who also observed a decreased amount of CO_2_ produced in the gases with increasing pyrolysis temperature.

The largest decrease in the CO_2_ content in the produced gases is observed when the temperature increases from 600 to 700 °C. This means that the complex mixture of proteins, lipids, polysaccharides and other components contained in sewage sludge is relatively thermally resistant. Its thermolability is induced at higher temperatures and exhibits characteristics of a typically endothermic reaction, proceeding more slowly at lower temperatures.

**Figure 4:**
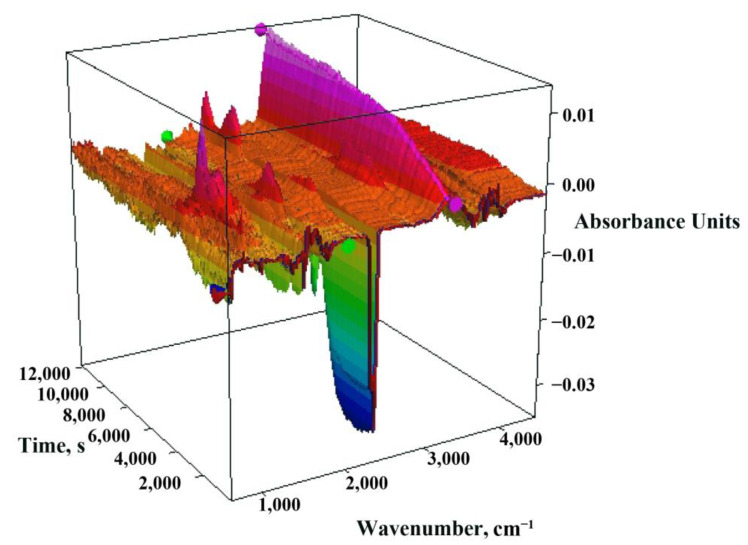
Three-dimensional FTIR spectra of evolved gases from the pyrolysis process of municipal sewage sludge.

These observations are confirmed by the three-dimensional FTIR spectrum presented in Figure 4 obtained during the thermal decomposition of the tested sewage sludge. The test was carried out in a nitrogen atmosphere, and the rate at which heat was applied to the sample was analogous to the heating rate during the pyrolysis process, that is, 10 °C·min^−1^. The picture of the spectrum clearly shows that the gases that evolved in the largest amounts were carbon oxides: CO_2_ (2400–2250 cm^−1^) and CO (2200–2000 cm^−1^) [45], methane (1306 and 1354 cm^−1^) [43], and smaller amounts of volatile organic and inorganic substances such as: alcohols (3584–3700 cm^−1^ and 1420–1330 cm^−1^) [46], amines (3500 cm^−1^) [47], volatile sulphur compounds (1415–1380 cm^−1^) [48] and alcohols and phenols (980–950 cm^−1^) [43].

**Figure 5:**
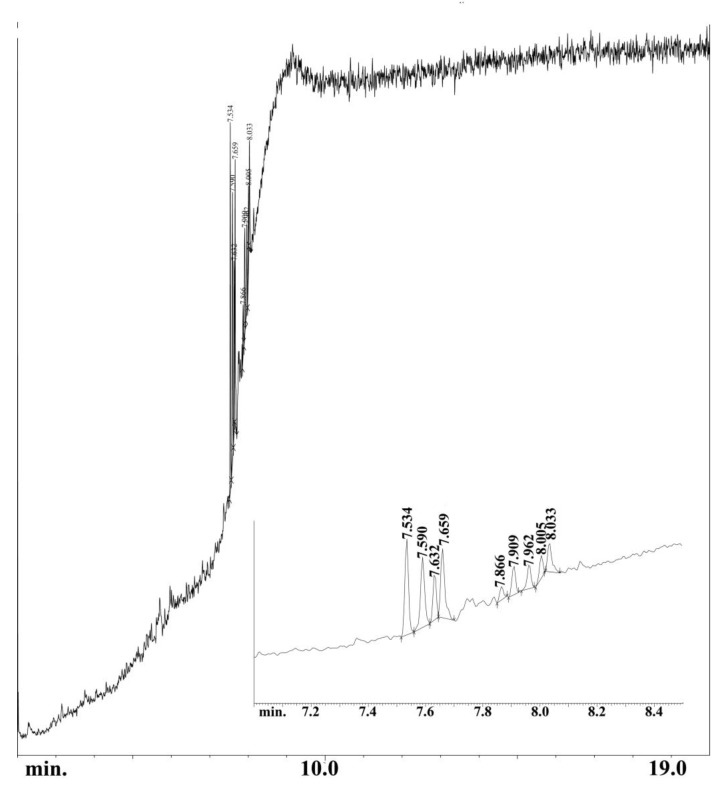
GC-MS chromatogram of the municipal sewage sludge tar.

Under the tested pyrolysis conditions, the tar yield was relatively low. The quantity of liquid products produced fluctuated slightly depending on the temperature and ranged from 32% to 40%. Changes in the amount of tar produced may have resulted from the condensation process of decomposition products in the tar before the organic bonds broke. Figure 5 shows an exemplary GC-MS chromatogram obtained for tar at 700 °C. In the composition of the mixture, mainly cholesterol derivatives as well as polycyclic and aliphatic siloxanes were identified. Among them, 5-beta-cholestan-3-alpha-ol and hexamethylcyclotrisiloxane were found—Table 5.

The amount of biochar produced and its composition depend largely on the temperature of the process. Table 6 shows the results of the carbon, hydrogen and nitrogen analysis. These data show that the percentage of carbon in the obtained product is not high and does not exceed 35%. The carbon content in the biochar decreases with increasing temperature. The same is true of the content of the other determined elements, that is, nitrogen and hydrogen. On the other hand, the data analysis clearly shows that extending the pyrolysis time has little effect on the elemental composition of the biochar. The degree of carbonisation expressed as the H/C molar ratio, according to the data in Table 6, clearly indicates that the increase in the temperature of the pyrolysis process causes its systematic decrease. At 400 °C, the H/C molar ratio is relatively high (0.7–0.9), which may indicate that the obtained biochar contains organic residues and is poorly charred. However, in the case of biochar obtained at a temperature of 700 °C, the value of the molar ratio drops to 0.3 and is close to the values characterising active carbons [49,50]. At temperatures above 600 °C, a clear decrease in the nitrogen content in the biochar is also visible, proving that only above this temperature do the bonds of nitrogen with organic residues begin to break.

These observations are confirmed by the data presented in Table 7. The technical analysis of the obtained biochar has shown that it is characterised by significant mineral content. The ash content of biochar ranges from 55% to 71% and rises with increasing process temperature. The increase in temperature also results in a decrease in the volatile matter content (from 18% to 0.4%) and slight changes in the moisture content. The consequence of reducing the carbon content in biochar is, of course, a decrease in its heat of combustion. The highest heat of combustion is characteristic of biochar obtained at a temperature of 400 °C (13 kJ·g^−1^), and the lowest at a temperature of 700 °C (9 kJ·g^−1^).

**Figure 6:**
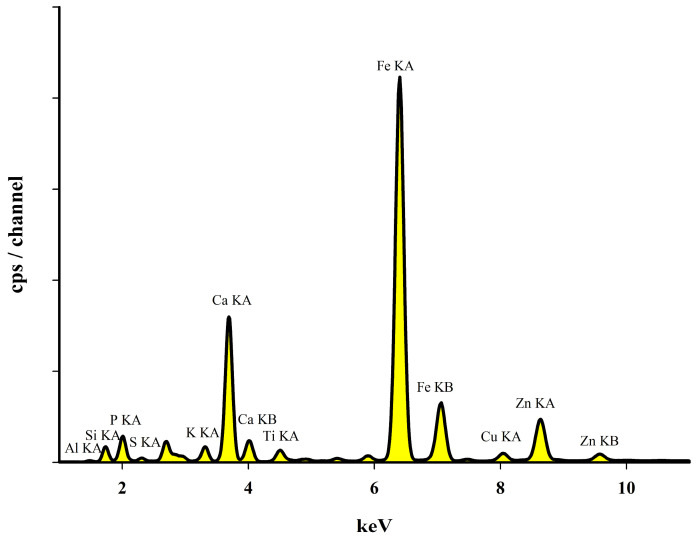
EDXRF spectrum of an example ash.

The ash analysis performed using the X-ray fluorescence (EDXRF) technique (Figure 6) showed that the obtained ash contains macro elements such as: Ca, Fe, Si, P, K, Mg or Al. However, it also contains quite large amounts of elements such as: S, Ti, Cr, Mn, Cu, Zn and Na.

**Figure 7:**
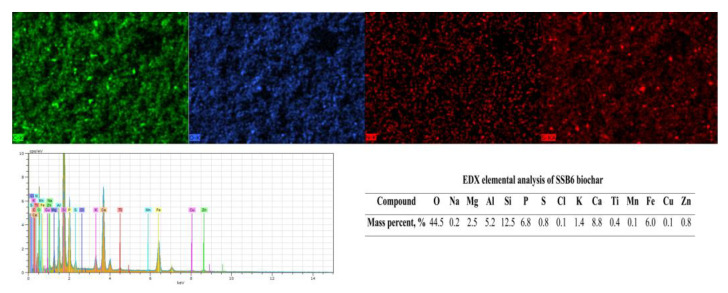
SEM-EDX analysis and mapping results of SSB6 biochar.

The conducted scanning electron microscopy (SEM) and energy-dispersive X-ray (EDX) analyses showed significant differences in the content of mineral components in the obtained biochars, the content of which rises with the increase of the process temperature. The presence of metals such as Ca, K, Na and Mg in the biochar is beneficial to the adsorption process. They can exchange or precipitate with sorbates and reduce their availability. Lu et al. [51] and Park et al. [52] observed that the adsorption efficiency according to this mechanism of heavy metals for rice straw and sludge biochar was 51.8% Pb^2+^ and 62.3% Cu^2+^, respectively. The exemplary mapping images of the carbon material presented in Figure 7 show that the distribution of elements in the biochar is uniform across its surface, which can ensure efficient sorption.

**Figure 8:**
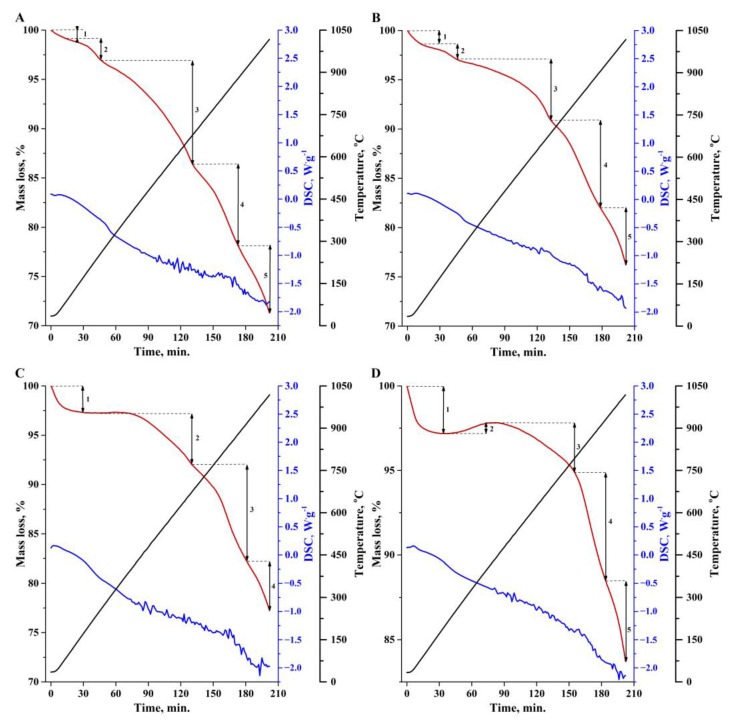
TGA-DTA analysis results of SSB1 (**A**), SSB4 (**B**), SSB5 (**C**) and SSB6 (**D**); (**−**) TGA, (**−**) DTA, (**−**) Temperature.

Thermogravimetry differential thermal analysis (TG-DTA) of the biochar samples was carried out in a nitrogen atmosphere at a flow rate of 100 cm^3^·min^−1^, with a heating rate of 5 °C·min^−1^. The appropriate TG-DTA curves are presented in Figure 8. Four or five stages can be distinguished in the thermal decomposition of the samples. The first three endothermic stages are associated with the loss of adsorption moisture and the decomposition of oxygen functional groups: carboxyl, hydroxyl and lactone. In the next two main stages of decomposition, the carbon skeleton of the sample, as well as nitrogen and sulphur-containing groups, are destroyed.

**Figure 9:**
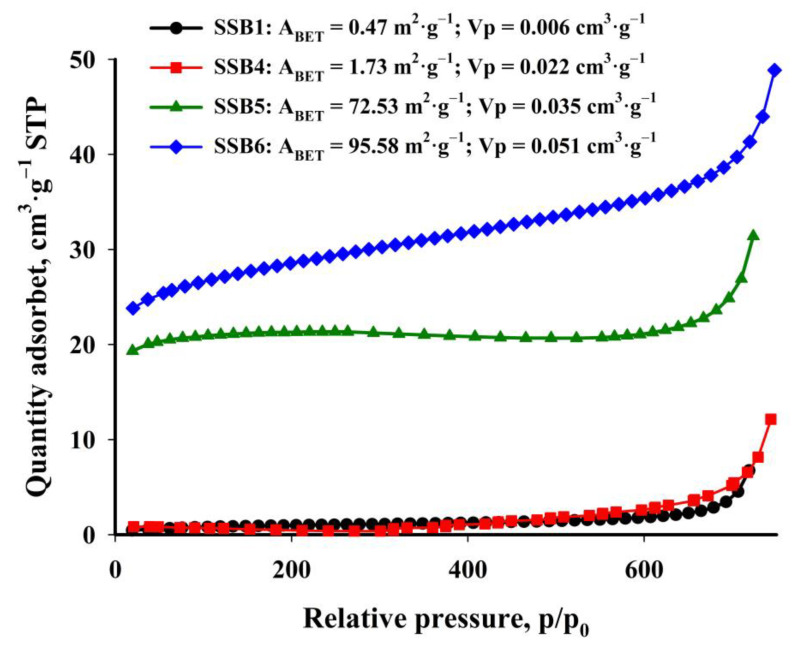
N_2_ adsorption isotherm of selected studied biochars.

Brunauer–Emmett–Teller (BET) surface area analysis has shown that the obtained biochar is characterised by a moderately developed specific surface (Figure 9). Biochar obtained at temperatures between 400 °C and 500 °C is a non-porous material with a specific surface area of less than 10 m^2^·g^−1^. Extending the time of the pyrolysis process does not increase the porosity of the material. Only at a temperature above 500 °C does the process of releasing volatile substances and creating open pores under the influence of heating accelerate. The specific surface initially increases to 73 m^2^·g^−1^ at 600 °C, and reaches its maximum at the highest tested temperature of 700 °C: 96 m^2^·g^−1^. An increase in the temperature of the pyrolysis process also results in a higher pore volume. However, it should be emphasised that the specific surface area of the obtained biochars is smaller than that of biochars obtained from other plant waste products. The studied samples contained heavy hydrocarbons, the degree of removal that can control the surface area’s size. The reason for this phenomenon may also be the blocking of pores by the metal oxide crystals present on the surface of the biochar. Li et al. [53], who obtained modified MgO biochar from sugar cane harvest residues, have confirmed this. They found that the surface area of their biochar decreased from 118 m^2^·g^−1^ to 27 m^2^·g^−1^ with an increase in MgO content from 2% to 20%.

**Figure 10:**
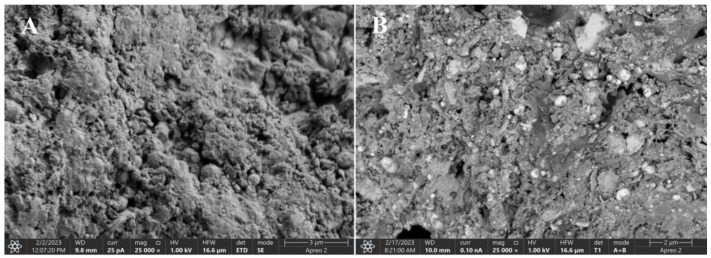
High-resolution scanning electron microscope images of SSB1 (**A**) and SSB6 (**B**) biochars.

The SEM images of the SSB1 and SSB6 sorption materials presented in Figure 10 confirm these observations. SSB1 has a practically flat surface, while SSB6 consists of particles of irregular shape and grain sizes that tend to agglomerate. The outer surface of the SSB6 also has deep fissures that can form after the removal of volatile organic matter during pyrolysis.

**Figure 11:**
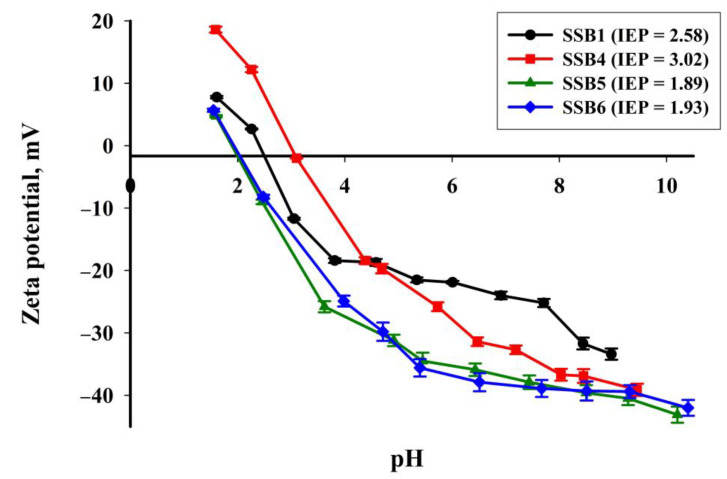
Zeta potential vs. pH for the obtained selected biochars.

Figure 11 shows the electrokinetic curves estimated for selected samples of the obtained carbon materials. All four samples are characterised by the fairly similar character of the curves and a wide range of zeta potential values, which depend strictly on pH. The presented curves are characteristic of carbon materials [54,55]. SSB1 has a zeta potential of 7.78–(−33.4) mV, SSB4 has 18.6–(−39.1) mV, SSB5 has 4.72–(−43.1) mV and SSB6 material has 5.68–(−42.0) mV, respectively in the analysed pH range. The surface of SSB5 and SSB6 materials is usually negatively charged above pH 2, while that of SSB1 and SSB4 is usually negatively charged only above pH 3. This phenomenon may be conducive to the effective binding of positively charged metal ions. The isoelectric points (pH where the zeta potential is 0) are 2.58, 3.03, 1.89 and 1.93 for SSB1, SSB4, SSB5 and SSB6, respectively.

### 3.4. Adsorption Tests

**Figure 12:**
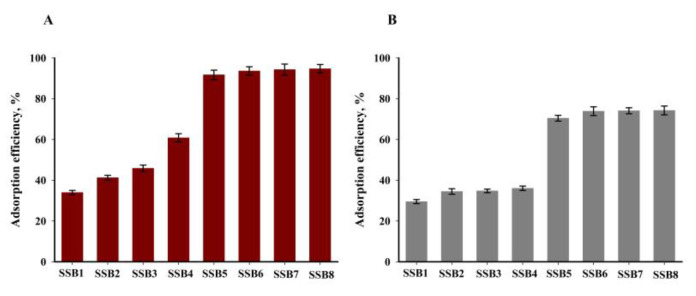
The results of adsorption of Cu^2+^ (**A**) and Zn^2+^ (**B**) on various sorbents (*T* = 30 °C, *t* = 240 min, *c* = 100 mg·dm^−3^, pH = 5, mass of adsorbent = 6.67 g·dm^−3^).

The test results presented in Chapter 3.3 clearly suggest that biochars obtained at temperatures of ≥600 °C are characterised by the best physicochemical properties by far in terms of sorption capacity. To confirm this, preliminary tests were carried out to determine the sorption properties of copper(II) and zinc(II) ions from aqueous solutions. The test results are shown in Figure 12. The tests were carried out for a solution of Cu^2+^ and Zn^2+^ ions at a concentration of 100 mg·dm^−3^. Biochars SSB5–8 are characterised by the best sorption properties in relation to both tested ions. The obtained efficiency of the adsorption process for SSB5 biochar is higher by about 30% than the value obtained for SSB4 for both tested ions. The presented data also indicate that extending the time of the pyrolysis process does not significantly improve the sorption capacity of the obtained biochars. Therefore, further tests were conducted for SSB6.

**Figure 13:**
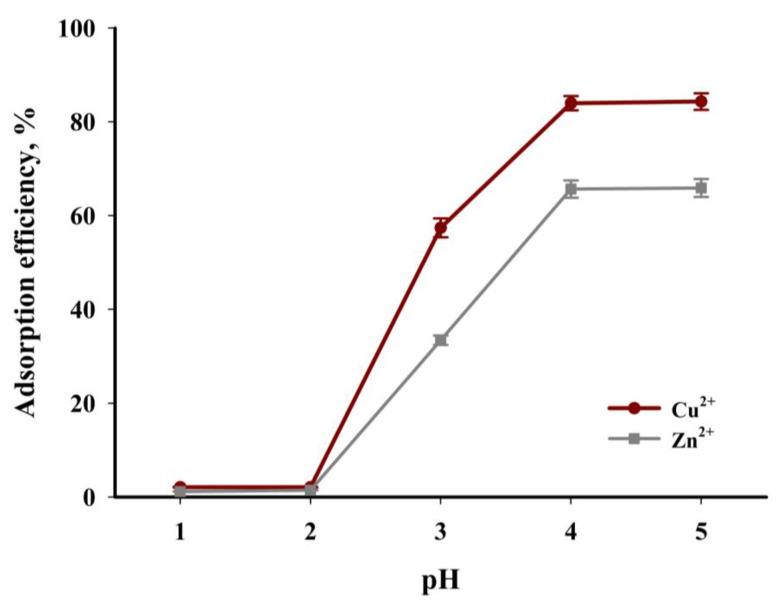
The influence of aqueous solution pH on adsorption efficiency (*T* = 30 °C, *t* = 240 min, *c* = 200 mg·dm^−3^, mass of adsorbent = 6.67 g·dm^−3^).

The next stage of the study involved determining the influence of pH on the degree of adsorption of Cu^2+^ and Zn^2+^ ions. It is an important parameter in the process of ion adsorption on a solid adsorbent, also due to technological solutions. The results of the tests determining the effect of the pH of the solution on the adsorption efficiency of Cu^2+^ and Zn^2+^ ions on the tested biochar are shown in Figure 13. The course of the points clearly shows that the adsorption of copper(II) and zinc(II) ions is clearly worse in a strongly acidic environment than in solutions with a higher pH. The calculated difference in adsorption process efficiency between pH = 1 and pH = 5 is 80%, and 65% for Cu^2+^ and Zn^2+^, respectively. Metal sorption in a strongly acidic environment is less efficient owing to the protonation of functional groups (that is, carboxylate, -COOH; and hydroxyl, -OH) on the sorbent surface, which undoubtedly reduces metal ion adsorption on the biochar surface due to electrostatic repulsion [56,57]. At low pH, hydronium ions present in higher concentrations also compete with metal ions for adsorption sites available on the biochar surface. An increase in the pH of the solution changes the charge on the surface of the biochar, creating more negatively charged groups and decreasing the competition of Cu^2+^ and Zn^2+^ ions and protons for active sites. It should be emphasised that Cu(OH)_2_ precipitation may occur when the pH of the solution exceeds 6, dominating the adsorption at a sufficiently high pH. In addition, such an environment generates the formation of copper(II) hydroxocomplexes Cu(OH)^+^, which can make complexation with surface functionalities present in biochar, consequently leading to a decrease in adsorption efficiency [58]. The analysis of the relationships presented in Figure 13 shows that the tested adsorbent has a much higher affinity for copper(II) ions than for zinc(II) ions.

**Figure 14:**
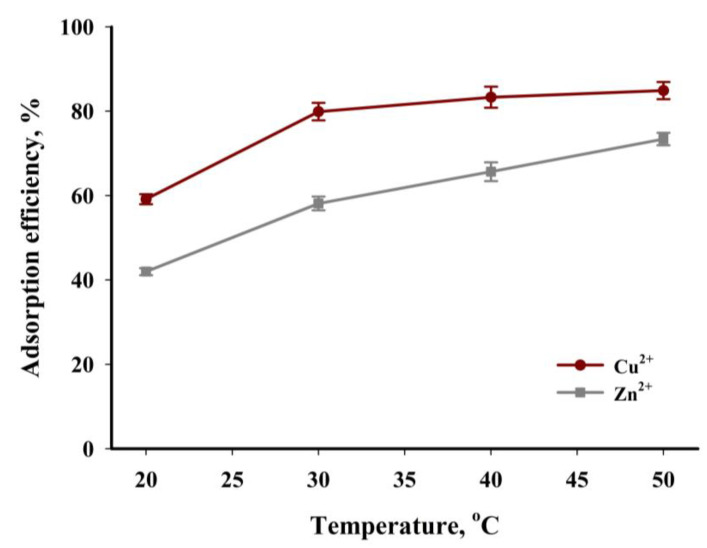
The influence of the studied process temperature on the adsorption efficiency (*t* = 240 min, *c* = 200 mg·dm^−3^, mass of adsorbent = 6.67 g·dm^−3^, pH = 5).

Another important parameter affecting the efficiency of the adsorption process is the process temperature. The analysis of the data presented in Figure 14 shows that an increase in the temperature of the tested process results in an increase in efficiency for both cations tested. The lowest efficiency was obtained for the experiments carried out at 20 °C: 60% and 42% for copper(II) and zinc(II) ions, respectively. The highest adsorption efficiency of Cu^2+^ (85%) and Zn^2+^ (73%) was obtained at 50 °C. The course of the points in Figure 14 shows that the temperature of the process has a greater impact on the adsorption process of zinc(II) ions than of copper(II) ions. In addition, it can be stated that the tested process is endothermic. A rise in the process temperature increases the kinetic energy of the tested ions, which is conducive to an increase in their mobility and spontaneous adsorption. An increase in temperature also causes a decrease in the viscosity of aqueous solutions, leading to an increase in the diffusion rate of ions toward the surface of the tested biochar. Many of the previously published papers found that the sorption process of metal cations on biochars has an endothermic nature, and the adsorption capacity increased with the increase in temperature [59,60].

**Figure 15:**
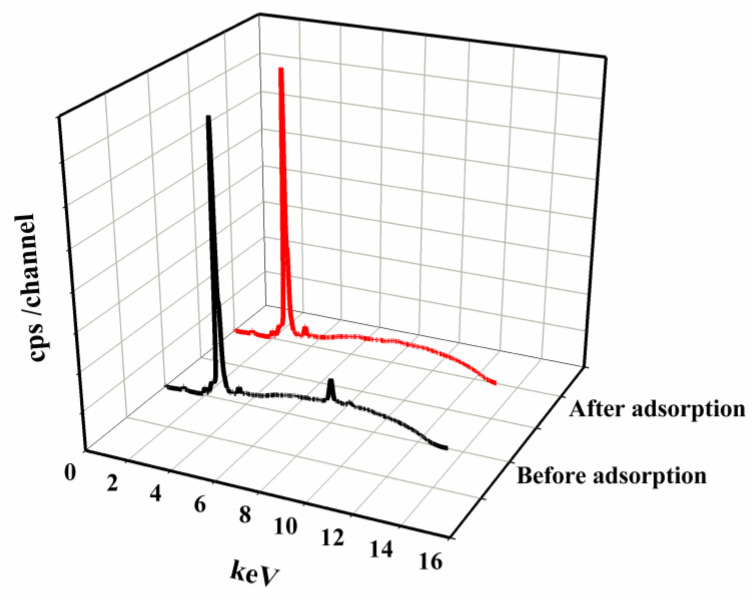
The EDXRF qualitative analysis of the solution before and after the adsorption process.

Figure 15 shows the energy-dispersive X-ray fluorescence (EDXRF) spectra of the solutions obtained before and after the Cu^2+^ ion adsorption process. According to the EDXRF theory, the characteristic radiation of an element appears in the spectrum at a certain excitation current [38]. The high peak at 2.696 keV, characteristic of both spectra, belongs to the material from which the Rh anti-cathode of the lamp is made. The spectrum of the solution before adsorption also shows two peaks at 8.040 and 8.904 keV: these are the Kα and Kβ characteristic lines for copper. The characteristic lines at the excitation current 2.013 and 3.690 can also be identified as the Kα lines of phosphorus and calcium, respectively. After the adsorption process, the copper signal disappears from the spectrum and the intensity of the line characteristic of calcium increases. A higher concentration of this element ion in the solution after the adsorption process may indicate that the adsorption process of copper(II) and zinc(II) ions on SSB6 is to some extent also based on the ion exchange process. This mechanism is consistent with the results obtained by other authors. For example, based on qualitative research, Cheng et al. determined that the adsorption mechanism on the biochar they developed was 90% based on precipitation and ion exchange [61]. The absence of characteristic lines for other elements included in the obtained biochar in the solution after adsorption indicates that the obtained sorbent is stable under the tested conditions and the sorbent components do not decompose in the solution during the adsorption process.

### 3.5. Adsorption Kinetics

**Figure 16:**
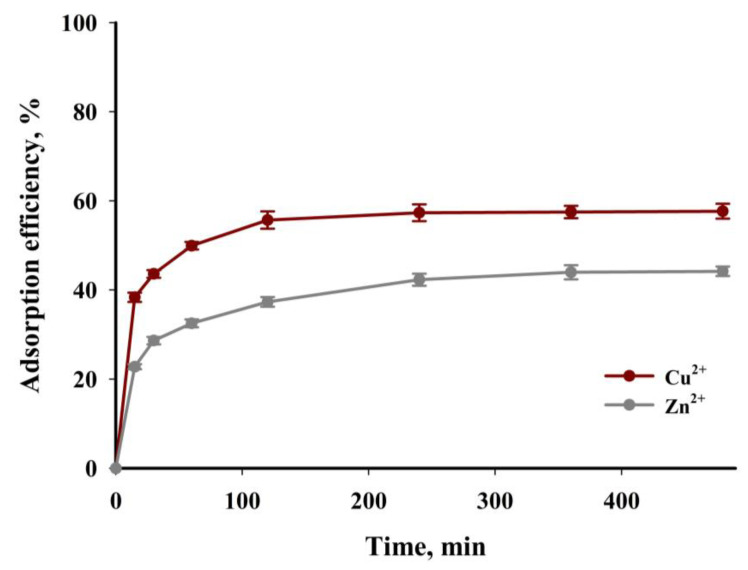
Adsorption efficiency as a function of time (*c* = 300 mg·dm^−3^, *T* = 40 °C, mass of adsorbent = 6.67 g·dm^−3^, pH = 5).

Figure 16 shows the results of experimental tests aimed at determining the impact of the time of the adsorption process on the efficiency of the process in relation to both tested cations. The tests were carried out for standard solutions with initial concentrations of copper(II) and zinc(II) cations amounting to 300 mg∙dm^−3^. The course of the points in Figure 16 shows that a high degree of removal of the tested ions is already achieved after the first hour of contact between the adsorbent and the adsorbate. The efficiency of the adsorption process after this contact time was 50% and 33% for Cu^2+^ and Zn^2+^, respectively. Initially, a significant increase in the adsorption of copper(II) and zinc(II) cations can be attributed to the presence of numerous unsaturated adsorption sites and functional groups on the biochar’s surface. A reduction in the number of free sites and functional groups causes the subsequent slow sorption. The equilibrium state for both considered cations is established relatively slowly in the tested system, namely after about four hours of the process. The time required to achieve equilibrium in the tested systems is not surprising. Previous research results have clearly demonstrated that, in terms of kinetics, metal ion adsorption is much slower on carbon sorbents than on inorganic sorbents and ion exchange resins [62,63,64].

Based on the presented results of experimental studies, appropriate kinetic models were calculated that could be used to design the adsorption process precisely. Adsorption kinetics of Cu^2+^ and Zn^2+^ cations on the examined biochar were analysed based on the kinetic equations of pseudo-first-order (PFO), pseudo-second-order (PSO) and intramolecular diffusion (IPD). Table 8 presents the calculated parameters of the kinetic equations.

The data in Table 8 clearly show that the pseudo-second-order kinetic model best describes the kinetics of the Cu^2+^ and Zn^2+^ cation adsorption process on the studied SSB6. This is confirmed by the very high linear correlation coefficients (0.999) obtained. Furthermore, the calculated adsorption capacity values have a strong correlation with the experimental data. The results may suggest that the process of adsorption of Cu^2+^ and Zn^2+^ cations on SSB6 is dominated by chemisorption, limited to some extent by covalent interactions. The calculated values of the constant k_2_ also indicate that the sorption of the Zn^2+^ cation was two times slower than the sorption of Cu^2+^. Moreover, the presented IPD model shows that the adsorption process of the tested cations proceeds in two stages. External diffusion and adsorption occur on the surface during the first stage. The second, much slower stage, is associated with adsorption on the pores’ surface and diffusion inside the pores.

### 3.6. Adsorption Isotherms

Three commonly used isotherm models describing experimental data were used to determine the adsorption mechanism and capacity of the SSB6 adsorbent: Langmuir, Freundlich and Temkin.

**Figure 17:**
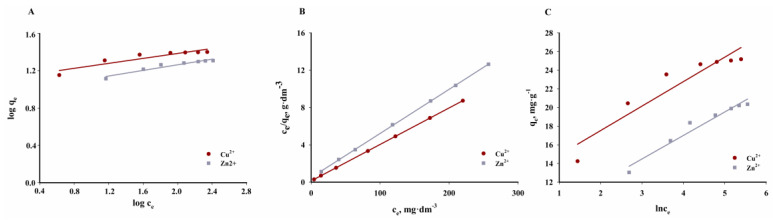
Freundlich (**A**), Langmuir (**B**), and Temkin (**C**) adsorption isotherms of Cu^2+^ and Zn^2+^ on SSB6 biochar (*T* = 40 °C, *t* = 240 min, mass of adsorbent = 6.67 g∙dm^−3^, pH = 5).

Table 9 summarises the calculated isotherm parameters and the values of R^2^, root mean square error (*RMSE*) and chi-square test ($\chi^{2}$). The graphical interpretation of the isotherms under consideration is shown in Figure 17.

According to the data presented in Table 9 and Figure 17, the adsorption process of Cu^2+^ and Zn^2+^ cations on the examined biochar is best described by the Langmuir isotherm. The *R*^2^ values obtained for the Freundlich and Temkin models are significantly lower than those obtained for the Langmuir isotherm. On the other hand, the calculated *RMSE* and *χ*^2^ values are higher. Therefore, SSB6 is characterised by a differentiated surface, and during the adsorption process, reactions occur mainly in the monolayer and there are no additional interactions that could give the effects of multilayer adsorption. The maximum sorption capacity of the biochar obtained from the sewage sludge in relation to the Cu^2+^ cation is 25.55 mg∙g^−1^, and in the case of Zn^2+^ it is 21.15 mg∙g^−1^.

### 3.7. Comparison with Other Sorbents and Cations

Table 10 and Table 11 compare the obtained maximum adsorption capacities in relation to Cu^2+^ and Zn^2+^ cations with the results obtained for various types of carbon sorbents.

Comparing the data presented in Table 10 and Table 11 reveals that the sorbent produced by the pyrolysis of sewage sludge has a relatively good sorption capacity for Cu^2+^ and Zn^2+^ ions.

### 3.8. The Proposed Sorption Mechanism

**Figure 18:**
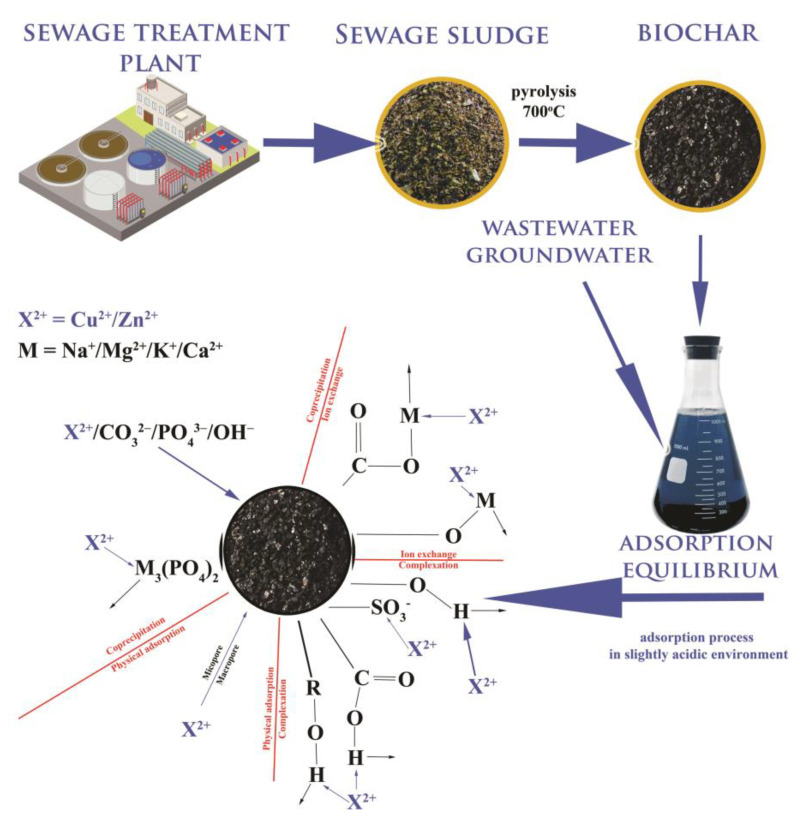
Proposed adsorbent/adsorbate interactions mechanism for obtained municipal sewage sludge biochar.

The mechanism of adsorbent/adsorbate interactions was proposed based on the experimental data and dependencies (the characteristics, adsorption kinetics, and isotherm results): Figure 18. The interactions appear to be chemical and electrostatic in nature, and are primarily affected by the pH of the reaction environment. SSB6 might mainly adsorb the Cu(II) and Zn(II) from an aqueous environment by oxygen-containing groups of hydrophilic sites (chemisorption), precipitation and porous structure of the adsorbent (physisorption) with minor contributions from ion exchange. The proposed mechanism is consistent with the findings of other authors [61,66,71].

## 4. Conclusions

This article describes the influence of the parameters of the municipal sewage sludge pyrolysis process on the quantity and properties of the obtained products. The proposed method of managing this municipal waste makes it possible to obtain a sorption material with relatively high sorption parameters.

The presented test results indicate that the main decomposition of the material took place at temperatures ranging from 180 to 700 °C. Analyses of the chemical composition of the gas showed that it contains mainly carbon(IV) oxide, carbon(II) oxide, methane and some volatile organic compounds, such as aldehydes, acids, alcohols and phenols. The content of carbon oxides in the gas decreased with increasing temperature, while the amount of methane produced increased. The gas obtained under high-temperature conditions can be used successfully to generate heat or energy.

The tar yield was relatively small and ranged around 30%. The GC-MS analysis of tar made it possible to identify mainly compounds of anthropogenic origin, such as cholesterol derivatives. As valuable components that could be recovered have not been identified in the composition of the tar, the management of the obtained tar may pose some environmental problems. An optional solution seems to be to subject the obtained tar to a destruction process through its combustion with excess oxygen.

With the increase in the process temperature, the yield of the obtained biochar decreased. Increasing the temperature of the pyrolysis process significantly improves its physicochemical properties and sorption capacity. Extending the time of the pyrolysis process does not significantly affect the quantity or quality of the obtained products.

The biochar obtained at 700 °C was characterised by the most favourable properties. The carbon material obtained under these conditions has a specific surface area (*A_BET_*) of nearly 100 m^2^·g^−1^ and a pore volume (*V_p_*) of 0.05 cm^3^·g^−1^. It is characterised by a relatively high affinity for Cu^2+^ and Zn^2+^ cations, which has been confirmed by experimental results. The adsorption process is largely dependent on the contact time of the adsorbate with the adsorbent, adsorption temperature, initial cation concentration and pH of the solution. The PSO model best describes the adsorption kinetics of Cu^2+^ and Zn^2+^ cations on biochar obtained from sewage sludge. Similarly, the Langmuir isotherm model better describes the mechanism of sorption of both tested cations on the obtained biochar, as evidenced by the higher linear correlation coefficient (*R*^2^ > 0.999) and low *RMSE* and *χ*^2^ values. The maximum adsorption capacity, calculated based on the Langmuir isotherm, is 26 mg·g^−1^ and 21 mg·g^−1^, respectively for copper(II) and zinc(II) cations.

An important benefit of the study was that it determined the parameters of the sewage sludge pyrolysis process to obtain a cheap and efficient biosorbent for water and technological wastewater treatment. The obtained biochar is characterised by relatively good sorption properties and can be used successfully in technological processes. It can also be the basis for obtaining surface-modified and high-performance carbon materials. For this purpose, however, it is necessary to add another kind of waste to the pyrolysis process (for example, mineral-poor agricultural waste) to increase the carbon content of the product.

## Figures and Tables

**Table 1:** Conditions of the pyrolysis process and product abbreviations used.

Condition	SSB1	SSB2	SSB3	SSB4	SSB5	SSB6	SSB7	SSB8
Temperature, °C	400	400	400	500	600	700	700	700
Time, h	1	3	5	1	1	1	3	5

**Table 2:** Content of selected inorganic components in sewage sludge.

Element Content	P	K	Ca	Si	Mg	Na	Fe	Al	Zn	Cu	Ti	Cr	Ni	Cd	Pb
		%									mg·kg^−1^				
This study	6.57	0.37	0.27	1.90	0.93	0.38	2.24	0.89	0.21	431	396	173	126	11	39
[43]	3.77	1.56	2.35	2.63	0.92	0.21	1.89	1.14	0.37	9700	1300	930	409	-	-

**Table 3:** The influence of pyrolysis condition on the products yields.

Process	Char	Tar	Gas
	%		
SSB1	57.0	31.9	11.1
SSB2	56.9	32.0	11.1
SSB3	56.7	32.1	11.2
SSB4	48.9	37.2	13.9
SSB5	45.7	38.6	15.7
SSB6	44.3	38.8	16.9
SSB7	43.9	39.2	16.9
SSB8	43.6	39.6	16.8

**Table 4:** Pyrolysis gas composition (N_2_ free).

Process	Composition of the Evolved Gases, mol%			
	CO_2_	CO	O_2_	CH_4_
SSB1	78.49	3.80	5.53	12.18
SSB4	76.76	2.65	4.35	16.24
SSB5	73.40	1.37	4.81	20.42
SSB6	67.37	0.81	3.54	28.29

**Table 5:** Semi-quantitative composition of tar (GC-MS analysis) collected in the condensation stages during gasification of SSB6.

Component	Normalised Peak Area, %	Retention Time, min
5-.beta.-cholestan-3.alpha.-ol, propionate (C_30_H_52_O_2_)	22.95	7.534
1,2-Dimethoxy-4-(1,3-dimethoxy-1-propenyl)benzene (C_13_H_18_O_4_)	18.47	7.590
5-.beta.-cholestan-3.alpha.-ol, octanoate(C_35_H_62_O_2_)	9.49	7.632
5-.beta.-cholestan-3.alpha.-ol, butyrate (C_31_H_54_O_2_)	18.87	7.659
Cyclotrisiloxane (C_6_H_18_O_3_Si_3_)	4.07	7.866
Silicic acid, (C_10_H_28_O_4_Si_3_)	7.03	7.909
1,2-Benzisothiazol-3-amine (C_13_H_20_N_2_SSi)	6.15	7.962
1,2-Benzisothiazol-3-amine (C_13_H_20_N_2_SSi)	5.16	8.005
Methyltris(trimethylsiloxy)silane (C_10_H_30_O_3_Si_4_)	7.82	8.033

**Table 6:** The influence of time and temperature of the pyrolysis process on the CHN elemental analysis results.

Biochar	Element, %			H/C Ratio
	N	C	H	
SSB1	4.01	32.53	2.38	0.88
SSB2	4.05	33.54	1.82	0.65
SSB3	4.02	32.56	1.80	0.66
SSB4	3.65	30.60	1.44	0.57
SSB5	2.79	27.45	0.86	0.38
SSB6	1.86	27.32	0.66	0.29
SSB7	1.72	27.42	0.64	0.28
SSB8	1.66	27.49	0.63	0.28

**Table 7:** Proximate analysis of biochars.

Biochar	Moisture	Ash	Volatiles	Heat of Combustion
	%			J·g^−1^
SSB1	1.1	54.7	17.9	13,272
SSB2	1.2	55.9	16.3	13,159
SSB3	0.8	56.2	15.3	13,065
SSB4	0.7	61.3	9.4	12,205
SSB5	0.6	68.6	3.2	10,414
SSB6	1.1	71.0	0.4	9550
SSB7	0.9	71.3	0.4	9530
SSB8	0.9	71.1	0.4	9550

**Table 8:** The kinetic parameters of Cu^2+^ and Zn^2+^ adsorption process onto the SSB6 biochar.

Kinetic Model	Parameters	Cation	
		Cu^2+^	Zn^2+^
	*q_e,exp_*, mg·g^−1^	24.8	20.3
PFO	*q_e,cal_*, mg·g^−1^	6.9	12.2
	*k*_1_, min^−1^	0.014	0.013
	*R* ^2^	0.943	0.988
PSO	*q_e,cal_*, mg·g^−1^	25.5	21.2
	*k*_2_, g·mg^−1^·min^−1^	0.005	0.002
	*R* ^2^	0.999	0.999
IPD	*k*_3_, mg·g^−1^·min^−1/2^	1.054	0.734
	*R* ^2^	0.979	0.953
	*k*_4_, mg·g^−1^·min^−1/2^	0.011	0.132
	*R* ^2^	0.898	0.852

*T* = 40 °C, *c* = 300 mg·dm^−3^, pH = 5, mass of adsorbent = 6.67 g·dm^−3^.

**Table 9:** Freundlich, Langmuir and Temkin isotherms parameters for adsorption of Cu^2+^ and Zn^2+^ cations onto SSB6.

Isotherm Model	Cu(II)	Zn(II)
1. Freundlich		
*K_F_*, mg·g^−1^	13.177	9.183
n	7.519	6.640
*R* ^2^	0.850	0.934
*RMSE*	1.820	0.712
*χ* ^2^	0.790	0.135
2. Langmuir		
*q_m_*, mg·g^−1^	25.55	21.15
*K*, dm^3^·mg^−1^	0.303	0.096
*R* ^2^	1.000	0.999
*RMSE*	0.164	0.366
*χ* ^2^	0.007	0.047
3. Temkin		
*A_T_*, dm^3^·g^−1^	108.010	15.709
*B*, J·mol^−1^	2.624	2.518
*R* ^2^	0.888	0.957
*RMSE*	1.479	0.601
*χ* ^2^	0.559	0.106

*T* = 40 °C, *t* = 240 min, pH = 5, mass of adsorbent = 6.67 g·dm^−3^.

**Table 10:** Different adsorbents’ capacities for removing Cu^2+^ cations from aqueous solutions.

Adsorbent	*q_m_* (mg·g^−1^)	Reference
Biochar	52.2	[65]
Hydrochar	48.2	[66]
Activated carbon	36.6	[67]
SSB6	25.6	This study
Manure biochar	21.9	[59]
Biochar	15.7	[68]
Sunflowers husks biochar	13.2	[69]
Paddy husk biochar	10.3	[70]
Hardwood biochar	4.39	[71]

**Table 11:** The ability of various adsorbents to remove Zn^2+^ cations from aqueous solutions.

Adsorbent	*q_m_* (mg∙g^−1^)	Reference
Activated carbon	100.8	[72]
Rice straw biochar	38.6	[51]
Biochar	35.8	[73]
Biochar	29.0	[65]
SSB6	21.2	This study
Corn straw biochar	11.0	[74]
Biochar	10.4	[68]
Paddy husk biochar	6.5	[70]
Hardwood biochar	2.31	[71]

## Data Availability

The authors confirm that the data supporting the findings of this study are available within the article.

## References

[1] Sim S., Ryu D. (2023). Effect of the concrete slurry waste ratio on supercritical CO_2_ sequestration. Materials.

[2] Lutyński M., Kielar J., Gajda D., Mikeska M., Najser J. (2023). High-pressure adsorption of CO_2_ and CH_4_ on biochar—A cost-effective sorbent for in situ applications. Materials.

[3] Wang R., Lu M., Wang J. (2022). Co-utilization of sewage sludge and rice husk in ceramsite preparation with selective adsorption capacity to Pb. Materials.

[4] Liang Y., Xu D., Feng P., Hao B., Guo Y., Wang S. (2021). Municipal sewage sludge incineration and its air pollution control. J. Clean. Prod..

[5] Antonkiewicz J., Popławska A., Kołodziej B., Ciarkowska K., Gambuś F., Bryk M., Babula J. (2020). Application of ash and municipal sewage sludge as macronutrient sources in sustainable plant biomass production. J. Environ. Manag..

[6] Gao N., Kamran K., Quan C., Williams P.T. (2020). Thermochemical conversion of sewage sludge: A critical review. Prog. Energy Combust. Sci..

[7] Yuan S., Dai X. (2017). Sewage sludge-based functional nanomaterials: Development and applications. Environ. Sci. Nano.

[8] Rulkens W. (2008). Sewage sludge as a biomass resource for the production of energy: Overview and assessment of the various options. Energy Fuels.

[9] Kominko H., Gorazda K., Wzorek Z. (2017). The possibility of organo-mineral fertilizer production from sewage sludge. Waste Biomass Valor..

[10] Szołdrowska D., Smol M. (2022). Agricultural use of municipal sewage sludge in the face of the European Green Deal challenges. Przem. Chem..

[11] Buta M., Hubeny J., Zieliński W., Harnisz M., Korzeniewska E. (2021). Sewage sludge in agriculture—The effects of selected chemical pollutants and emerging genetic resistance determinants on the quality of soil and crops—A review. Ecotoxicol. Environ. Saf..

[12] Eurostat. https://ec.europa.eu/eurostat/databrowser/view/ten00030/default/table?lang=en.

[13] Xu J., Ping L., Cao H., Liu W., Gu Y., Lin X., Huang J. (2019). Application status of so-processing municipal sewage sludge in cement kilns in China. Sustainability.

[14] Mukawa J., Pająk T., Rzepecki T., Banaś M. (2022). Energy potential of biogas from sewage sludge after thermal hydrolysis and digestion. Energies.

[15] Collivignarelli M.C., Abbà A., Miino M.C., Torretta V. (2019). What advanced treatments can be used to minimize the production of sewage sludge in WWTPs?. Appl. Sci..

[16] Cucina M., De Nisi P., Sordi S., Adani F. (2021). Sewage sludge as N-fertilizers for crop production enabling the circular bioeconomy in agriculture: A Challenge for the New EU Regulation 1009/2019. Sustainability.

[17] Lewandowski W., Januszewicz K., Kosakowski W. (2019). Efficiency and proportions of waste tyre pyrolysis products depending on the reactor type—A review. J. Anal. Appl. Pyrolysis.

[18] Jiang S., Sheng G., Jiang H. (2019). Advances in the characterization methods of biomass pyrolysis products. ACS Sustain. Chem. Eng..

[19] Belbessai S., Azara A., Abatzoglou N. (2022). Recent advances in the decontamination and upgrading of waste plastic pyrolysis products: An overview. Processes.

[20] Li Y., Zhao H., Sui X., Wang X., Ji H. (2022). Studies on individual pyrolysis and co-pyrolysis of peat–biomass blends: Thermal decomposition behavior, possible synergism, product characteristic evaluations and kinetics. Fuel.

[21] Zhang Q., Bu T., Lu J., Jin Z., Chen D., Qiu F., Feng Y., Hu W. (2022). Effects of quicklime conditioning on the volatile reforming and tar elimination performance of sewage sludge pyrochar. J. Anal. Appl. Pyrolysis.

[22] Yu F., Hu Y., Li L., Guo Q., Zhu Y., Jiao L., Wang Y., Cui X. (2022). Investigation on oxygen-controlled sewage sludge carbonization with low temperature: From thermal behavior to three-phase product properties. Environ. Sci. Pollut. Res..

[23] Wang B., Liu Y., Guan Y., Feng Y. (2022). Characteristic of the production of hydrogen-rich combustible gas by pyrolysis of high-ash sewage sludge. J. Clean. Prod..

[24] Gerasimov G.Y., Khaskhachikh V.V., Sychev G.A., Larina O., Zaichenko V. (2022). Study of a two-stage pyrolytic conversion of dried sewage sludge into synthesis gas. Russ. J. Phys. Chem. B.

[25] Foong S.Y., Liew R.K., Yang Y., Cheng Y.W., Mahari W.A.W., Peng W., Lam S.S. (2020). Valorization of biomass waste to engineered activated biochar by microwave pyrolysis: Progress, challenges, and future directions. Chem. Eng. J..

[26] Nie T., Yang X., Chen H., Muller K., Shaheen S.M., Rinklebe J., Song H., Xu S., Wu F., Wang H. (2021). Effect of biochar aging and co-existence of diethyl phthalate on the mono-sorption of cadmium and zinc to biochar-treated soils. J. Hazard. Mater..

[27] Wang J., Wang S. (2021). Preparation, modification and environmental application of biochar: A review. J. Clean. Prod..

[28] Mazurek K., Weidner E., Drużyński S., Ciesielczyk F., Kiełkowska U., Wróbel-Kaszanek A., Jesionowski T. (2020). Lanthanum enriched TiO_2_-ZrO_2_ hybrid material with tailored physicochemical properties dedicated to separation of lithium and cobalt(II) raising from the hydrometallurgical stage of the recycling process of lithium ion batteries. Hydrometallurgy.

[29] Mazurek K., Drużyński S., Kiełkowska U., Bielicka A., Gluzińska J. (2023). Application of sulphate and magnesium enriched waste rapeseed cake biochar for recovery of Cu(II) and Zn(II) from industrial wastewater generated in sulphuric acid plants. Hydrometallurgy.

[30] Shang H., Li Y., Liu J., Wan Y., Yu Y.L. (2020). Preparation of nitrogen doped magnesium oxide modified biochar and its sorption efficiency of lead ions in aqueous solution. Bioresour. Technol..

[31] Yin G., Tao L., Chen X., Bolan N.S., Sarkar B., Lin Q., Wang H. (2021). Quantitative analysis on the mechanism of Cd^2+^ removal by MgCl_2_-modified biochar in aqueous solutions. J. Hazard. Mater..

[32] Wołowicz A., Hubicki Z. (2022). Removal of vanadium by ion exchange resins from model and real solutions from spent V_2_O_5_ catalyst. Hydrometallurgy.

[33] Xiang Y., Ding S., Chen X., Cao C., Sun J., Xu L., Liu G. (2020). Recovery of gold from waste solutions using a new RFB resin. Hydrometallurgy.

[34] Lingamdinne L.P., Koduru J.R., Roh H., Choi Y., Chang Y., Yang J. (2016). Adsorption removal of Co(II) from waste-water using graphene oxide. Hydrometallurgy.

[35] Qiu X., Hu H., Yang J., Wang C., Cheng Z. (2018). Removal of trace copper from simulated nickel electrolytes using a new chelating resin. Hydrometallurgy.

[36] Mei X., Cui Z., Sheng Q., Zhou J., Li C. (2023). Application of the improved POA-RF model in predicting the strength and energy absorption property of a novel aseismic rubber-concrete material. Materials.

[37] Frezzini M.A., Massimi L., Astolfi M.L., Canepari S., Giuliano A. (2019). Food waste materials as low-cost adsorbents for the removal of volatile organic compounds from wastewater. Materials.

[38] Brouwer P. (2010). Theory of XRF.

[39] Wang J., Liu H., Deng H., Jin M., Xiao H., Yao H. (2020). Deep dewatering of sewage sludge and simultaneous preparation of derived fuel via carbonaceous skeleton-aided thermal hydrolysis. Chem. Eng. J..

[40] Ruiz-Hernando M., Martinez-Elorza G., Labanda J., Llorens J. (2013). Dewaterability of sewage sludge by ultrasonic, thermal and chemical treatments. Chem. Eng. J..

[41] Maw M.M., Boontanon N., Fujii S., Boontanon S.K. (2022). Rapid and efficient removal of organic matter from sewage sludge for extraction of microplastics. Sci. Total Environ..

[42] Żukowska G., Mazurkiewicz J., Myszura M., Czekała W. (2019). Heat energy and gas emissions during composting of sewage sludge. Energies.

[43] Gao N., Li J., Qi B., Li A., Duan Y., Wang Z. (2014). Thermal analysis and products distribution of dried sewage sludge pyrolysis. J. Anal. Appl. Pyrolysis.

[44] Djandja O.S., Wang Z., Wang F., Xu Y., Duan P. (2020). Pyrolysis of municipal sewage sludge for biofuel production: A review. Ind. Eng. Chem. Res..

[45] Ma Z., Chen D., Gu J., Bao B., Zhang Q. (2015). Determination of pyrolysis characteristics and kinetics of palm kernel shell using TGA–FTIR and model-free integral methods. Energy Convers. Manag..

[46] Afzal M., Khan A.S., Zeshan B., Riaz M., Ejaz U., Saleem A., Zaineb R., Sindhu H.A., Yean C.Y., Ahmed N. (2023). Characterization of bioactive compounds and novel proteins derived from promising source citrullus colocynthis along with in-vitro and in-vivo activities. Molecules.

[47] Yadav A., Kumar A., Sharma K., Shukla M. (2019). Investigating the effects of amine functionalized graphene on the mechanical properties of epoxy nanocomposites. Mater. Today: Proc..

[48] Liang P., Qin X., Bai G., Wu Z., Sun D., Zhang Y., Jiao T. (2019). Effects of ionic liquid pretreatment on pyrolysis characteristics of a high-sulfur bituminous coal. Fuel.

[49] Ozcimen D., Karaosmanoglu F. (2004). Production and characterization of bio-oil and biochar from rapeseed cake. Renew. Energy.

[50] Chen B.L., Zhou D.D., Zhu L.Z. (2008). Transitional adsorption and partition of nonpolar and polar aromatic contaminants by biochars of pine needles with different pyrolytic temperatures. Environ. Sci. Technol..

[51] Lu H., Zhang W., Yang Y., Huang X., Wang S., Qiu R. (2012). Relative distribution of Pb^2+^ sorption mechanisms by sludge-derived biochar. Water Res..

[52] Park J.H., Wang J.J., Kim S.H., Cho J.S., Kang S.W., Delaune R.D., Han K.J., Seo D.C. (2017). Recycling of rice straw through pyrolysis and its adsorption behaviors for Cu and Zn ions in aqueous solution. Colloids Surf. A.

[53] Li R., Wang J.J., Zhou B., Awasthi M.K., Ali A., Zhang Z., Lahori A.H., Mahar A. (2016). Recovery of phosphate from aqueous solution by magnesium oxide decorated magnetic biochar and its potential as phosphate-based fertilizer substitute. Bioresour. Technol..

[54] Samsuri A.W., Sadegh-Zadeh F., Seh-Bardan B.J. (2013). Adsorption of As(III) and As(V) by Fe coated biochars and biochars produced from empty fruit bunch and rice husk. J. Environ. Chem. Eng..

[55] Song G., Guo Y., Li G., Zhao W., Yu Y. (2019). Comparison for adsorption of tetracycline and cefradine using biochar derived from seaweed Sargassum sp.. Desalin. Water Treat..

[56] Zhang P., Zhang X., Yuan X., Xie R., Han L. (2021). Characteristics, adsorption behaviors, Cu(II) adsorption mechanisms by cow manure biochar derived at various pyrolysis temperatures. Bioresour. Technol..

[57] Katiyar R., Patel A.K., Nguyen T., Singhania R.R., Chen C., Dong C. (2021). Adsorption of copper(II) in aqueous solution using biochars derived from Ascophyllum nodosum seaweed. Bioresour. Technol..

[58] Khandaker S., Hossain T., Saha P., Rayhan U., Islam A., Choudhury T., Awual M.R. (2021). Functionalized layered double hydroxides composite bio-adsorbent for efficient copper(II) ion encapsulation from wastewater. J. Environ. Manag..

[59] Meng J., Feng X., Dai Z., Liu X., Wu J., Xu J. (2014). Adsorption characteristics of Cu(II) from aqueous solution onto biochar derived from swine manure. Environ. Sci. Pollut. Res..

[60] Liu Z., Zhang F. (2009). Removal of lead from water using biochars prepared from hydrothermalliquefaction of biomass. J. Hazard. Mater..

[61] Cheng S., Zhao S., Guo H., Xing B., Liu Y., Zhang C., Ma M. (2022). High-efficiency removal of lead/cadmium from wastewater by MgO modified biochar derived from crofton weed. Bioresour. Technol..

[62] Ben-Ali S., Jaouali I., Souissi-Najar S., Quederni A. (2017). Characterization and adsorption capacity of raw pomegranate peel biosorbent for copper removal. J. Clean. Prod..

[63] Maleki A., Hajizadeh Z., Sharifi V., Embadi Z. (2019). A green, porous and eco-friendly magnetic geopolymer adsorbent for heavy metals removal from aqueous solutions. J. Clean. Prod..

[64] Bouhamed F., Elouear Z., Bouzid J., Ouddane B. (2015). Multi-component adsorption of copper, nickel and zinc from aqueous solutions onto activated carbon prepared from date stones. Environ. Sci. Pollut. Res..

[65] Mazurek K., Drużyński S., Kiełkowska U., Szłyk E. (2021). New separation material obtained from waste rapeseed cake for copper(II) and zinc(II) removal from the industrial wastewater. Materials.

[66] Semercioz A.S., Gogus F., Celekli A., Bozkurt H. (2017). Development of carbonaceous material from grapefruit peel with microwave implemented-low temperature hydrothermal carbonization technique for the adsorption of Cu(II). J. Clean. Prod..

[67] Wang B., Lan J., Bo C., Gong B., Ou J. (2023). Preparation of Ganoderma lucidum bran-based biological activated carbon for dual-functional adsorption and detection of copper ions. Materials.

[68] Cibati A., Foereid B., Bissessur A., Hapca S. (2017). Assessment of Miscanthus × giganteus derived biochar as copper and zinc adsorbent: Study of the effect of pyrolysis temperature, pH and hydrogen peroxide modification. J. Clean. Prod..

[69] Tomczyk A., Sokołowska Z., Boguta P. (2020). Biomass type effect on biochar surface characteristic and adsorption capacity relative to silver and copper. Fuel.

[70] Wijeyawardana P., Nanayakkara N., Gunasekara C., Karunarathna A., Law D., Pramanik B. (2022). Removal of Cu, Pb and Zn from stormwater using an industrially manufactured sawdust and paddy husk derived biochar. Environ. Technol. Innov..

[71] Jiang S., Huang L., Nguyen T., Ok Y.S., Rudolph V., Yang H., Zhang D. (2016). Copper and zinc adsorption by softwood and hardwood biochars under elevated sulphate-induced salinity and acidic pH conditions. Chemosphere.

[72] Zhang X., Hao Y., Wang X., Chen Z. (2017). Rapid removal of Zinc(II) from aqueous solutions using a mesoporous activated carbon prepared from agricultural waste. Materials.

[73] Sanyang L., Ghani W.A.W.A.K., Idris A., Mansor A. (2014). Zinc removal from wastewater using hydrogel modified biochar. Appl. Mech. Mater..

[74] Chen X., Chen G., Chen L., Chen Y., Lehmann J., McBride M.B., Hay A.G. (2011). Adsorption of copper and zinc by biochars produced from pyrolysis of hardwood and corn straw in aqueous solution. Bioresour. Technol..

